# Draft Genome Sequence of a Bacteroides fragilis Strain Isolated from Peritoneal Fluid of a Patient from Kazakhstan

**DOI:** 10.1128/MRA.00377-20

**Published:** 2020-06-25

**Authors:** Elena Zholdybayeva, Saniya Kozhakhmetova, Sabina Atavliyeva, Pavel Tarlykov, Yerlan Ramankulov

**Affiliations:** a National Center for Biotechnology, Nur-Sultan, Kazakhstan; University of Maryland School of Medicine

## Abstract

Here, we report the draft genome sequence of Bacteroides fragilis strain KZ02, isolated from a patient with peritonitis hospitalized in a health facility in Nur-Sultan, Kazakhstan. The genome of the strain contains 4,103 protein-coding genes, including the *cepA* gene, which causes resistance to beta-lactam antibiotics.

## ANNOUNCEMENT

The Bacteroides genus, including the Bacteroides fragilis group, dominates in intra-abdominal anaerobic infections (1). The B. fragilis group consists of 24 species, including B. fragilis, B. vulgatus, B. ovatus, B. thetaiotaomicron, B. uniformis, B. caccae, and Parabacteroides distasonis (2). B. fragilis has a capsular polysaccharide complex and a virulence factor, which enhance the pathogenic properties of the bacterium (3). Differences in rates of resistance to some antibiotics were detected among the *Bacteroides* species and between isolates from different clinical sources (4).

Here, we sequenced the genome of B. fragilis strain KZ02 using an Ion Torrent Personal Genome Machine (PGM) sequencing platform (Thermo Fisher). The B. fragilis strain KZ02 was isolated from a patient with peritonitis hospitalized in Nur-Sultan, Kazakhstan. Informed consent and questionnaires were approved by the Local Ethics Committee of the RSE National Center for Biotechnology of the Ministry of Education and Science of the Republic of Kazakhstan (extract from protocol no. 4 of 29 August 2017). Organisms were collected using swabs from wounds from the drainage and subsequent immersion of probes in a tube containing Amies medium. Bacterial cultures were cultivated on bile esculin agar (BEA) in anaerobic medium (in anaerobic jars using the Gas-pak method) at 37°C for 72 h. B. fragilis strain KZ02 was identified using 16S rRNA gene sequence analysis and the MALDI Biotyper microbial identification system (Bruker). The stocks of the strain were stored at −70°C in a cryoprotective medium. DNA was extracted using the traditional cetyltrimethylammonium bromide (CTAB) procedure (5). The whole-genome shotgun (WGS) sequence data were retrieved with the Ion Torrent sequencing platform as described previously (6). DNA libraries were prepared using an Ion Xpress Plus fragment library kit (Thermo Fisher). An Ion PGM HiQ sequencing kit and an Ion 318 Chip v.2 BC (both Thermo Fisher) were used for sequencing. The complete genome of B. fragilis strain KZ02 has a length of 5,148,042 bp. The genome of B. fragilis was sequenced with 33-fold coverage, a mean read length of 252 bp, and a G+C content of 43.09%. A total of 844,759 reads were produced. The quality check of the reads was done using FastQC v.0.11.9 (7). A total of 89 contigs, with an *N*_50_ value of 254,934 bp, were *de novo* assembled using SPAdes v.3.1.0 (8). The final assemblies were annotated with the NCBI Prokaryotic Genome Annotation Pipeline v.4.10 (9). Default parameters were used for all software. Annotation of the KZ02 genome with the NCBI Prokaryotic Genome Annotation Pipeline (PGAP) identified a total of 4,503 genes, 4,103 of which predicted coding sequences, including the *cepA* gene, which causes resistance to beta-lactam antibiotics. In addition, 61 tRNAs and 1 complete 16S rRNA were detected and predicted to be responsible for the bacterium’s pathogenicity and transmission patterns.

### Data availability.

This whole-genome shotgun project has been deposited at DDBJ/ENA/GenBank under the accession no. SSKK01000000. The raw data from BioProject no. PRJNA531645 were submitted to the NCBI SRA under experiment accession no. SRX5656190.

## References

[1] Wexler HM 2007 Bacteroides: the good, the bad, and the nitty-gritty. Clin Microbiol Rev 20:593–621. doi:10.1128/CMR.00008-07. 17934076 PMC2176045

[2] Schuetz AN 2014 Antimicrobial resistance and susceptibility testing of anaerobic bacteria. Clin Infect Dis 59:698–705. doi:10.1093/cid/ciu395. 24867792

[3] Jotwani R, Gupta U, Watanabe K, Ueno K 1992 Pathogenicity of *Bacteroides fragilis* group in rat intra-abdominal abscesses. Microbiol Immunol 36:1041–1049. doi:10.1111/j.1348-0421.1992.tb02108.x. 1479960

[4] Nagy E, Urban E, Nord CE 2011 Antimicrobial susceptibility of *Bacteroides fragilis* group isolates in Europe: 20 years of experience. Clin Microbiol Infect 17:371–379. doi:10.1111/j.1469-0691.2010.03256.x. 20456453

[5] Wilson K 2001 Preparation of genomic DNA from bacteria. Curr Protoc Mol Biol Chapter 2:Unit 2.4. doi:10.1002/0471142727.mb0204s56. 18265184

[6] Shevtsov A, Tarlykov P, Zholdybayeva E, Shevtsova E, Momynkulov D, Sytnik I, Karibaev T, Chsherbakov A, Momynaliev K 2013 Draft genome sequence of the live vaccine strain *Brucella abortus* 82. Genome Announc 1:e01101-13. doi:10.1128/genomeA.01101-13. 24371203 PMC3873613

[7] Andrews S 2019 FastQC: a quality control tool for high throughput sequence data. http://www.bioinformatics.babraham.ac.uk/projects/fastqc.

[8] Nurk S, Bankevich A, Antipov D, Gurevich AA, Korobeynikov A, Lapidus A, Prjibelski AD, Pyshkin A, Sirotkin A, Sirotkin Y, Stepanauskas R, Clingenpeel SR, Woyke T, McLean JS, Lasken R, Tesler G, Alekseyev MA, Pevzner PA 2013 Assembling single-cell genomes and mini-metagenomes from chimeric MDA products. J Comput Biol 20:714–737. doi:10.1089/cmb.2013.0084. 24093227 PMC3791033

[9] Tatusova T, DiCuccio M, Badretdin A, Chetvernin V, Nawrocki EP, Zaslavsky L, Lomsadze A, Pruitt KD, Borodovsky M, Ostell J 2016 NCBI Prokaryotic Genome Annotation Pipeline. Nucleic Acids Res 44:6614–6624. doi:10.1093/nar/gkw569. 27342282 PMC5001611

